# Warning to Travelers

**Published:** 1880-09

**Authors:** 


WARNING TO TRAVELERS.—A communication which we have received from a traveler describes a severe outbreak of typhoid fever in Switzerland, to be traced, it is stated, as most of such outbreaks are traced, to impure drinking water. This frequent cause of disease to travelers, will, in the end, scare away travelers to a considerable extent from Continental travels, unless the local authorities of the principal towns of summer-resort on the Continent manifest a more earnest determination to purify the air, soil, and water, and especially to provide a perfectly pure and undeniable source of drinking water, which is rarely to be found at present in any Continental town or village. Sir Henry Thompson, adverting to this abundant source of danger to travelers, recently recommended that every traveler should carry with him a filter and a teapot, by way of practically abolishing, by personal care, some of the danger of impure water by securing that it should be very thoroughly boiled before being used. Dr. Hermann Weber, whose experience of foreign resorts is perhaps greater than that of any other English authority, has published a similar warning to travelers, and has recommended them to use Apollinaris water whenever it is to be obtained as an undeniably pure drinking water, which would secure them from these dangers; and he has stated that he has known, in more than one instance, when members of the same traveling party have been careful to adopt this precaution, while others have neglected it, that those who adopted such precautions have been saved from typhoid fever, which attacked other members of the party. In the meanwhile, some such precaution for obtaining drinking water of absolute and guaranteed purity must recommend itself as a necessary means of safety. Recent analyses by chemical authorities, of which some of the results are before us, have shown that the water contained in the syphons which are introduced at foreign *restaurants* is not more reliable than the ordinary water supply; indeed, a table before us, to which, perhaps, we shall subsequently have to refer, indicates that, in one great foreign city at least, the water in the syphons is very much more impure than even the ordinary city drinking water, being in some cases little better than diluted sewage water. It appears that the manufacturers of these aërated waters in foreign syphons are by no means very careful from what kind of surface wells they draw their supply, or how they purify their water; and on the whole, the danger of drinking the aërated water of syphons is, unless the quality be definitely ascertained, greater even than that of drinking the ordinary impure water. It is quite time that foreign authorities should turn more serious attention to this subject. The evidence of the carriage of typhoid poison by contaminated water, overwhelming as the demonstration has been in this country, is by no means sufficiently appreciated abroad; indeed, the subject has been so imperfectly treated, that some foreign authorities profess absolute ignorance of facts which may now be taken as among the best established of modern times. It is well, until they have become more enlightened, that travelers should regard drinking water with precaution, and should be satisfied in some way or other that the table-water they drink is of absolute purity; and such assurance is best obtained by confining themselves, when traveling, to the use of a natural mineral water, suitable for table purposes and of undoubted pure origin.

—*British Medical Journal.*

